# Effectiveness of a Community Health Worker-Driven Intervention in Improving the Quality of Life of Caregivers of Children With Disability in Rural Karnataka, India

**DOI:** 10.7759/cureus.41798

**Published:** 2023-07-12

**Authors:** Sakthi Arasu, Deepthi N Shanbhag

**Affiliations:** 1 Occupational Health, St. John's Medical College, Bangalore, IND; 2 Community Health, St. John's Medical College, Bangalore, IND

**Keywords:** community trial, quasi-experimental study, community health worker intervention, quality of life, caregiver burden

## Abstract

Purpose

To assess the effectiveness of a community-health-worker (CHW)-driven intervention in improving the quality of life (QOL) of caregivers of children with disability in rural Karnataka, India.

Methodology

A community-based quasi-experimental study with cluster randomization on the village level was done. CHWs provided structured health education and training for the intervention arm. Pre- and post-intervention, the QOL and Zarit burden scores were compared between and within the two arms.

Results

From baseline, the physical domain score improved from 49.66 to 53.88 (p < 0.001). The Zarit burden scores decreased from 33.27 to 28.89 (p < 0.001). On comparing the post-test QOL scores between the two arms, the physical domain scores increased from 51.68 to 56.08 (p = 0.025). The Zarit burden scores also significantly decreased from 31.50 to 26.28.

Conclusion

The intervention by the CHWs on the caregivers has significant improvements in the physical domain of QOL and a reduction in caregiver burden.

## Introduction

Community health workers (CHWs) are members of the communities where they work, are answerable to the communities for their activities, are supported by the health system but not necessarily a part of its organization, and have shorter training than professional workers [1]. These CHWs proved to be a main arsenal in tackling healthcare delivery in both resourceful and resourceless facilities [2]. In well-funded, well-equipped, and well-staffed primary health centers (PHCs), CHWs' effects are multiplied. In areas where the health system is weak, these workers are the only sources of reaching out to the people, especially for maternal and child health (MCH). CHWs proved to be not just a stop-gap measure but an integral part of the healthcare professionals [3]. It has been shown that, for every 1$ invested in CHW-based activities, the return on investment is 2.47$ Medicaid [4].

Community-based rehabilitation (CBR) services for children with disability started in the year 2006 at the Community Health and Training Centre (CHTC), Mugalur (Bangalore District), Karnataka, India. As a part of the rehabilitation services, a multidisciplinary team assesses the children, and an intervention is planned. The service has not formally included the caregivers as a part of the holistic approach toward rehabilitation. It has been seen that improvement in the quality of life (QOL) of caregivers will also improve the QOL of children with disability [5].

Despite the large burden on child development, family life, and economics, research in the area of caregivers of disabled children is inadequate, especially from low- and middle-income countries. Services for caregivers will gain focus and importance in the country only with the recognition of the magnitude of the problems faced by them. There is a dearth of published studies, which have used community-based health workers as an interventional tool for improving the QOL through a home-based approach for the primary caregivers of disabled children.

Hence, this study aims to assess the effectiveness of a CHW-driven intervention in improving the QOL of caregivers and bringing down the caregiver burden.

## Materials and methods

A baseline assessment of the QOL of the caregivers was done using the WHOQOL-BREF questionnaire, the caregiver burden using the Zarit Burden Interview, and the children’s disability using WHODAS [6]. WHOQOL-BREF: (World Health Organization - Quality of Life) The interviewer administered the WHOQOL-BREF questionnaire to collect information on the QOL of the caregivers. Questions are about how they feel about QOL, health, and other aspects of life in the last four weeks. Scoring is done in four domains: physical, psychological, social, and environmental using the Likert scale. Domain scores are scaled in a positive direction (i.e. higher scores denote higher QOL). The mean score of items within each domain is used to calculate the domain score. Mean scores are then multiplied by four in order to make domain scores comparable with the scores used in WHOQOL-100. 

The Zarit caregiver burden scale was administered by the interviewer to assess the degree of caregiver burden. Responses are in ordinal variables, namely, 0: NEVER, 1: RARELY, 2: SOMETIMES, 3: QUITE FREQUENTLY, 4: NEARLY ALWAYS. Interpretation of scores is as follows: 0-21, little or no burden; 21-40, mild to moderate burden; 41-60, moderate to severe burden; and 61-88, severe burden. This showed that the mean caregiver burden according to the Zarit scale was 33.27, and mean burden scores were significantly different between the disability domains of the children. Mean QOL scores for each domain were 49.6 in physical, 60.47 in psychological, 45.67 in social, and 58.44 in environmental domains [6].

The current study was done as a quasi-experimental study with cluster randomization. The study area was the four PHC areas in rural Bangalore (Mugalur, Dommasandra, Anugondanahalli, Lakkur). The sampling unit was the primary caregivers of children with disabilities between the age groups of 0-18 years, registered in CBR services and residing in the area for at least two years in the four PHC areas.

The sample size was calculated to be 50 in each arm of the study with alpha at 5% and 80% power based on a standard deviation of 3.35 from a previous study [7]. We assumed a 30% improvement in the mean score for the QOL of caregivers of children with disability (from 13.7 to 17.81). We planned to sample from 42 clusters (each village is a cluster), with an intra-cluster correlation of 0.05. The sample size calculated was 50, which was taken as 50 caregivers for the control group and 50 caregivers for the intervention group.

**Figure 1:**
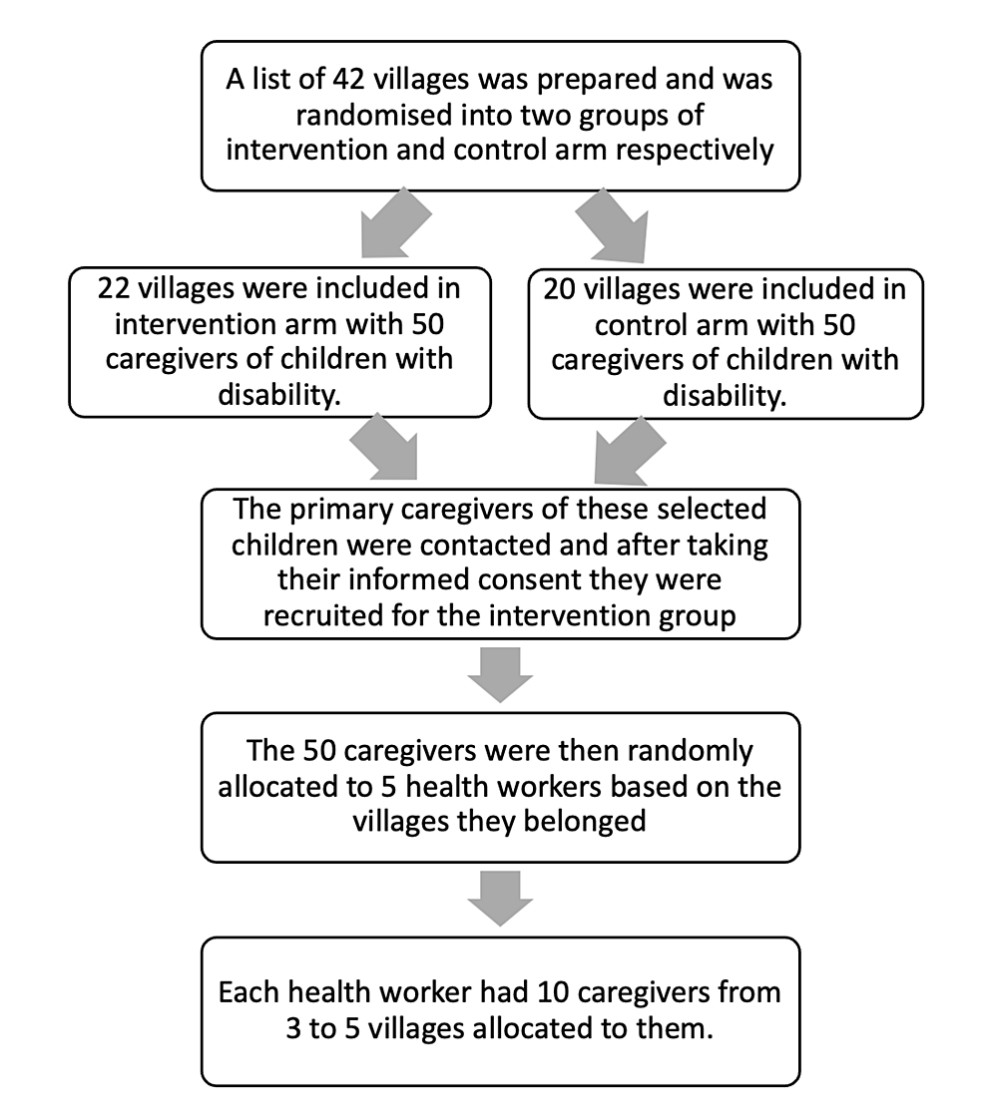
Process flow of the methodology in the cluster randomized experiment.

The villages under the four PHC areas from where the children were included in the baseline study [6] were listed. Each village was taken as a cluster. The population within the village cluster was as heterogeneous as possible, and there was homogeneity between the village clusters. The clusters were mutually exclusive and collectively exhaustive. A random sampling technique was used on relevant clusters to choose which clusters to include in the study. Each of the clusters was randomized into control and intervention groups (Figure 1).

We included primary caregivers of children with a disability under 18 years who are currently residing in the four PHC areas, and they should be permanent residents for the past two years. Primary caregivers who are not able to communicate due to severe speech and hearing impairment and intellectual impairment were excluded. An Institutional Ethical Committee approval was taken for this study.

The CHWs were randomly allocated 10 caregivers each. The randomization was at the village level (cluster), with approximately 4-5 villages per health worker, under which the 10 caregivers resided. Care was taken that the health worker did not t get to the village where she has been working for the past years. The health workers made a monthly visit and taught the caregivers about the importance of rest and nutrition, trained them on how to lift a bedridden child, and taught them about a balanced diet and sleep.

The intervention was divided into four parts.

Training of CHWs

Training modules were developed based on the “Caregiver Training Workbook” [8] by the Department of Veteran Affairs of the USA. This was validated and translated into the local language based on the already existing caregiver training modules. The training modules included the assessment of QOL, assessment of caregiver burden, and chapters on staying healthy, physical activity, nutrition, psychological good being, overcoming stress for the caregivers, and a checklist for health problems. Health education materials such as flipcharts and show cards were developed on good health practices and were translated into the local language. The CHWs underwent extensive training for five days with one-day field testing by experts in the field of social work and rehabilitation facilitated by the investigator. The CHWs were also trained to use health education materials and checklists when they go for home visits to ensure that the intervention is uniform across all health workers. The CHWs were given a follow-up register where they documented the date and proceedings of each visit.

Intervention by the CHWs with a home-based approach

Each of the five CHWs had four to five village clusters, under which 10 caregivers of the intervention arm were residing. The intervention was for a period of six months, with one visit a month by the health worker. The CHW proforma had a consent form, socio-demographic details of the caregiver, baseline QOL, and health problems checklist, which were administered to their allocated caregiver on the first visit. The health workers made regular home visits once a month for giving advice and counseling on nutrition, a balanced diet, training on exercises, how to lift a bedridden child, protecting the back, advice on proper rest, etc. The visits involved a wide range of health education sessions given to the caregivers using flipcharts, show cards, and demonstrations.

Monitoring of the home-based intervention by the principal investigator

The follow-up register of all five CHWs was verified and recorded every month by the investigator. The investigator monitored the intervention of each health worker by giving home visits to two randomly selected caregivers of each health worker. The investigator used a pre-tested supervision checklist tool to assess the quality of the intervention being given during a session by the health worker. These methods ensured that the quality of content and the delivery were adequate when the health worker delivered the intervention. A total of 10 such supervisory home visits were given two visits per health worker.

Post-intervention - QOL of both the control and intervention arms

After six months, the post-test QOL was assessed using the WHOQOL-BREF questionnaire [9] and the caregiver burden using the Zarit scale [10] in both the control and intervention groups by the investigator.

## Results

On comparing the caregiver characteristics from the intervention and control groups using chi-square tests for categorical variables and the Mann-Witney U test for the continuous variables, except for the mean age, every other factor was similar, and the groups were comparable (Table 1). Children’s mean disability assessment scale scores were not significantly different, showing that the different disability domains were equally distributed between the groups. The key outcome variables, namely, the QOL and the caregiver burden (ZBI) were not significantly different at baseline (Table 1).

**Table 1:** Comparison of various socio-demographic and other baseline factors between the intervention and control groups. *Mann-Whitney U test; # chi-square test

Serial	Factor		Control Group (Mean+/- SD or %) (n = 50)	Intervention Group (Mean+/- SD or %) (n = 50)	p-Value
1	Age		35.02 (12.04)	37.74 (12.23)	0.09*
2	Gender	Male	0 (0%)	3(100%)	0.242^#^
		Female	50 (51.5%)	47 (48.5%)	
3	Relation	Mother	43 (52.4%)	39 (47.6%)	0.42^#^
		Father	0 (0%)	3 (100%)	
		Grandmother	5 (45.4%)	6 (54.6%)	
		Others	2 (50%)	2 (50%)	
4	Income	<10,000	36 (50%)	36 (50%)	1.0^#^
		>10,000	14 (50%)	14 (50%)	
5	Religion	Hindu	46 (51.1%)	44 (48.9%)	0.505^#^
		Muslim	4 (40%)	6 (60%)	
6	Marital Status	Married	42 (51.8%)	39 (48.2%)	0.243^#^
		Widowed	4 (33.3%)	8 (66.7%)	
		Divorced	0 (0%)	3 (100%)	
		Single	1 (100%)	0 (0%)	
8	Type of Family	Nuclear	24 (46.1%)	28 (53.9%)	0.687^#^
		Joint	7 (58.3%)	5 (41.7%)	
		3 Generations	19 (52.7%)	17 (47.3%)	
9	Education	Uneducated	13 (44.8%)	16 (55.2%)	0.352^#^
		Primary	3 (60%)	2 (40%)	
		Middle	15 (50%)	15 (50%)	
		High	14 (46.6%)	16 (53.4%)	
		PU	4 (80%)	1 (20%)	
		Graduate	1 (100%)	0 (0%)	
10	Occupation	Housewife	31 (47.6%)	34 (52.4%)	0.339^#^
		Farming	7 (53.8%)	6 (46.2%)	
		Skilled	0 (0%)	2 (100%)	
		Semi-skilled	0 (0%)	1 (100%)	
		Unskilled	12 (63.1%)	7 (36.9%)	
11	Weight		51 (9.06)	53.34 (10.6)	0.351*
12	Blood Pressure	Normal	42 (48.8%)	44 (51.2%)	0.729^#^
		Hypertensive	8 (57.1%)	6 (42.9%)	
12	Disability Domains	Hearing Impairment	7 (63.6%)	4 (36.3%)	0.2^#^
		Locomotor Difficulties	10 (66.6%)	5 (33.3%)	
		Mental Retardation	18 (52.9%)	16 (47.1%)	
		Multiple Disabilities	14 (36.8%)	24 (63.2%)	
		Speech Problems	0	1 (100%)	
		Visual Impairments	1 (100%)	0	
13	WHOQOL-BREF	Physical	50.48 (8.09)	48.84 (10.82)	0.329*
		Mental	60.82 (8.89)	60.12 (8.89)	0.909*
		Social	46.60 (19.11)	44.74 (21.5)	0.687*
		Environmental	59.26 (14.77)	57.62 (14.83)	0.733*
14	Zarit Burden Scores		31.32 (11.87)	35.22 (13.92)	0.299*
15	WHODAS Scores		54.9 (22.3)	59.63 (26.51)	0.385*

After six months of intervention, end-line assessment with comparisons (Table 2) using the Mann-Whitney U test showed a significant increase from baseline in the physical domain of the QOL (49.66-53.88 at p < 0.001). Other domain scores of the QOL increased, but they were not significant. ZBI scores decreased from 33.27 to 28.89, and this reduction was significant (p < 0.001). Moreover, on comparing the intervention and control groups after intervention (Table 3), there were similar significant improvements in the physical domain of QOL and the ZBI.

**Table 2:** Quality of life and caregiver burden before and after the intervention in the study population. * Wilcoxon signed-rank test

Score		Before - Mean (SD)	After- Mean (SD)	p-Value
WHOQOL-BREF	Physical	49.66 (93.54)	53.88 (10.5)	<0.001*
	Mental	60.47 (8.85)	59.81 (8.38)	0.344*
	Social	45.67 (20.28)	47.04 (17.9)	0.467*
	Environmental	58.44 (14.75)	58.79 (12.60)	0.356*
Zarit Burden Scores		33.27 (13.03)	28.89 (11.10)	<0.001*

**Table 3:** Post-intervention comparison of quality of life and caregiver burden of the study population between intervention and control groups. *Mann-Whitney U test

Post-Test Scores		Control Group - Mean (SD)	Intervention Group - Mean (SD)	p-Value
WHOQOL-BREF	Physical	51.68 (8.8)	56.08 (11.8)	0.025*
	Mental	60.16 (8.72)	59.46 (8.1)	0.746*
	Social	49.72 (17.07)	44.36 (18.47)	0.121*
	Environmental	57.94 (11.72)	59.64 (13.4)	0.389*
Zarit Burden Scores		31.50 (11.8)	26.28 (9.7)	0.013*

We compared the QOL domain scores of the caregivers assigned to each health worker after the cluster randomization before and after the intervention (Table 4). The QOL domain scores were comparable at the baseline and after the intervention; the domain scores showed significant differences in the physical and social domains. This shows that the intervention and the subsequent improvements are also dependent on the health worker providing the intervention. 

**Table 4:** Differences in the WHOQOL scores of the caregivers trained by different health workers (numbered 1-5) before and after the intervention. *Kruskal-Wallis test

Variable		Physical Domain - Mean (SD)		Mental Domain - Mean (SD)		Social Domain - Mean (SD)		Environmental Domain - Mean (SD)	
		Pre	Post	Pre	Post	Pre	Post	Pre	Post
Health Workers	1	55.67 (9.57)	57.6 (11.63)	52 (10.4)	60.10 (7.52)	54.22 (14.38)	47.22 (13.33)	56.89 (12.4)	26.30 (8.3)
	2	47.3 (7.4)	52 (10.39)	63.22 (6.8)	55.56 (8.57)	48.67 (18.37)	36.9 (16.79)	57.78 (13.31)	24.89 (8.89)
	3	47 (9.4)	61.33 (10.68)	59.5 (8.5)	62.50 (8.42)	38.1 (16.5)	55.6 (13.07)	60.1 (13.9)	29 (5.90)
	4	49.7 (13.22)	61.22 (10.74)	60.8 (8.9)	60.56 (8.38)	46.8 (22.6)	49.89 (22.53)	60.9 (19.35)	26.89 (7.11)
	5	45.67 (12.02)	50.42 (12.49)	63.83 (5.9)	58.5 (7.76)	38.50 (29.23)	34.92 (18.87)	53.25 (15.381)	24.58 (15.11)
p-Value		0.313*	0.08*	0.157*	0.44*	0.385*	0.021*	0.731*	0.610*

ZBI scores did not show a similar pattern; both the pre- and post-intervention ZBI scores of caregivers were not significantly different between the health workers who delivered the intervention (Table 5).

**Table 5:** Differences in the Zarit burden scores of the caregivers trained by different health workers (numbered 1-5) before and after the intervention. *Kruskal-Wallis test

Health Worker	ZBI Before Intervention - Mean (SD)	p-Value	ZBI After Intervention - Mean (SD)	p-Value
1	31.32 (11.98)	0.458*	26.89 (7.11)	0.06*
2	36.89 (10.76)		26.30 (8.31)	
3	31.67 (13.21)		24.58 (15.11)	
4	38.90 (8.21)		24.89 (8.89)	
5	33.75 (20.19)		29.00 (5.981)	

## Discussion

Post-intervention QOL and ZBI

The average post-intervention QOL scores are 53.88 + 10.59 for the physical domain, 59.81 + 8.38 for the psychiatric domain, 47.04 + 17.9 for the social domain, and 58.79 +12.6 for the environmental domain. The post-intervention ZBI according to the Zarit scale showed 27% without burden, 59% with mild burden, 14% with moderate burden, and none with severe one. The mean Zarit score was 28.89 + 11.1.

On comparing the pre- and post-intervention QOL scores, the physical domain score improved from 49.66 + 9.54 to 53.88 + 10.5, and this was statistically significant (p < 0.001). Improvement in the physical domain of the QOL is because the intervention received by the caregivers for the six months was concentrated mostly on their physical health with regard to nutrition, rest, sleep, and back pain. One of the key components of intervention is how to handle and lift a disabled child, which was an important contributor to back pain. The other domain scores had minor changes, which were not statistically significant. The six-month duration is too less time to appreciate the significant changes in the psychological, social, and environmental domains of the QOL in caregivers.

The ZBI scores decreased from 33.27 + 13.03 to 28.89 + 11.1, which was statistically significant (p < 0.001). The training for caregivers also included counseling sessions and suggestions on how to overcome stress related to caregiving. Counseling sessions included one-to-one and group sessions where they talk about the difficulties faced in caregiving to their peers. Stress management exercises such as deep breathing and muscle relaxation techniques helped the caregivers to relax in the midst of their packed lives. The caregivers realized the importance of their health and well-being to have an impact on the health of the child, and as they started taking care of themselves, the perceived burden automatically came down.

In a meta-analysis done by Pinquart [11], of the 32 studies, which included the ZBI scores before and after the intervention, there was a mean reduction of 0.12, which corresponded to 53% of the caregivers having an above-average reduction in the burden. Another systemic review by Vernooij-Dassen [12] had a reduction of 0.14 among 12 studies. Our study showed a reduction of 4.38 in the ZBI score.

On comparing the post-test scores in the QOL between the intervention and control groups, there was a significant difference in the physical domain (p = 0.025) as the scores increased from 51.68(8.8) to 56.08(11.8). The ZBI scores were also significantly different, which decreased from 31.50 (11.8) to 26.28(9.7), which was statistically significant (p = 0.013). The QOL physical domain scores and the ZBI remained almost the same in caregivers who did not receive training. For those who underwent interventions through the health workers, the QOL and burden scores significantly improved compared to those who did not.

Intervention by the CHWs

The post-intervention QOL scores among the caregivers were compared between the different health workers who provided the intervention visits to them. Improvements in the social domain (p = 0.02) and physical domain (p = 0.08) were significantly different. The possible reason is the difference in communication skills of the health worker, the rapport building, and the ability to gain trust even in difficult circumstances, which may have played a role. The post-intervention ZBI scores of the caregivers, compared with the health workers who trained them, showed that the scores are not significantly different (p = 0.06).

The CHW-driven intervention is being used extensively across the globe for varying health services, from addressing post-partum depression [13], for support in diabetes for immigrants [14], care for childhood diseases in Liberia [15], increasing healthcare access in marginalized communities of New York [16], improve access to cancer care [17], increasing antenatal contacts and promoting institutional deliveries [18], and addressing unmet needs among children with asthma [19]. The CHW-driven intervention is cost-effective and can benefit the unreached population of healthcare [20].

The health workers in the study are working in the CHTC, Mugalur, for the past 10 years in services related to disability, along with eye and ear care. Some of these health workers are specialists in disability care and management at home. They were trained in community-based rehabilitation and are in charge of conducting monthly meetings for these caregivers in their in-charge villages. All these experiences would have enhanced the caregiver training of some health workers.

Recommendations

Mothers alone cannot take care of a child with special needs all through the day. Awareness should be created of the importance of caregiver health and the need for sharing the responsibility of caring for the child. The caregivers should be given regular health check-ups, with monitoring of blood sugar levels and blood pressure. Many mothers had to leave their job or stopped going to work to take care of the child, which took a toll on the finances of the family. They should be made aware of the schemes and monetary benefits available for the disabled child, which they can avail to take care of the health expenses of the child.

The domain of disability of the child has an influence on the QOL and caregiver burden. There should be special counseling sessions for the caregivers in regard to disability understanding and how to provide care. Some factors, which are associated with the QOL and can be modified, should be addressed, such as creating opportunities for caregivers to work from home (e.g., in small-scale industries that operate at household levels, such as cooking food for parcels, tying flowers for garlands, or tailoring) and providing education and training on how to care for the back of caregivers and regarding weight distribution when lifting and mobilizing the children.

Marital status is also an important factor associated with the QOL, and marriage counseling is a must for couples with disabled children right from childbirth so that they can support one another in caring for the child instead of blaming each other. The CHW-driven intervention should continue with the introduction of new training modules and periodic reinforcements for the health workers (i.e. training the trainers). The parents should be educated about special schools available for children with mental retardation, lower IQ, and learning disabilities; special schools with braille-based education for visually challenged children; and separate schools for hearing-impaired children. The informal caregivers in the family should be covered under health insurance, and steps should be taken to reach out to these caregivers and bring them under insurance coverage.

Limitations

The socio-demography could not be assessed in a structured manner because of hesitation in divulging sensitive information regarding income or not allowing us to see inside the house to assess their standard of living. The intervention period was six months owing to the time constraints, but this will be overcome by the continuous training of the caregivers by the health workers. Among the five health workers who were involved in the study, two of them already had prior training in community-based disability rehabilitation, which may have affected the training they gave to the caregivers.

## Conclusions

There was a significant increase in the physical domain scores and a decrease in the caregiver burden scores between the intervention and the control groups. Moreover, the health workers can be trained in different topics or given revision lessons regarding the interventions. The model of delivering health and well-being support to caregivers through community health workers will pave the way for a lot more interventions to reach the unreached. The intervention should be continued for longer periods to obtain significant changes in the other domains of the QOL. Reinforcement of the training and introduction of newer methods and modules in training is needed.
